# Pan-cancer analysis of the prognostic and immunological role of ANLN: An onco-immunological biomarker

**DOI:** 10.3389/fgene.2022.922472

**Published:** 2022-08-05

**Authors:** Kejun Liu, Lei Cui, Cunquan Li, Chaofeng Tang, Yiming Niu, Ji Hao, Yang Bu, Bendong Chen

**Affiliations:** ^1^ Department of Hepatobiliary Surgery, General Hospital of Ningxia Medical University, Yinchuan, China; ^2^ Ningxia Hepatobiliary and Pancreatic Surgical Diseases Clinical Medical Research Center, Yinchuan, China; ^3^ School of Clinical Medicine, Ningxia Medical University, Yinchuan, China; ^4^ Department of Hepatobiliary Surgery, People’s Hospital of Ningxia Hui Autonomous Region, Yinchuan, China

**Keywords:** pan-cancer, anillin actin-binding protein (ANLN), immunization, prognosis, bioinformatics analysis

## Abstract

Anillin actin-binding protein (ANLN) is crucially involved in cell proliferation and migration. Moreover, ANLN is significantly in tumor progression in several types of human malignant tumors; however, it remains unclear whether ANLN acts through common molecular pathways within different tumor microenvironments, pathogeneses, prognoses and immunotherapy contexts. Therefore, this study aimed to perform bioinformatics analysis to examine the correlation of ANLN with tumor immune infiltration, immune evasion, tumor progression, immunotherapy, and tumor prognosis. We observed increased ANLN expression in multiple tumors, which could be involved in tumor cell proliferation, migration, infiltration, and prognosis. The level of ANLN methylation and genetic alteration was associated with prognosis in numerous tumors. ANLN facilitates tumor immune evasion through different mechanisms, which involve T-cell exclusion in different cancer types and tumor-infiltrating immune cells in colon adenocarcinoma, kidney renal clear cell carcinoma, liver hepatocellular carcinoma, and prostate adenocarcinoma. Additionally, ANLN is correlated with immune or chemotherapeutic outcomes in malignant cancers. Notably, ANLN expression may be a predictive biomarker for the response to immune checkpoint inhibitors. Taken together, our findings suggest that ANLN can be used as an onco-immunological biomarker and could serve as a hallmark for tumor screening, prognosis, individualized treatment design, and follow-up.

## Introduction

Worldwide, cancer remains the leading cause of morbidity and mortality; moreover, it imposes huge health and economic burden on society (Sung et al., 2021). Current therapies mainly include surgery, radiotherapy, and immunotherapy; however, delayed diagnosis or distant metastasis impedes most patients from undergoing radical surgery (Siegel et al., 2021). Recently, immune checkpoint blockade (ICB) has been shown to be efficacious in immunotherapy for various cancer types. Recent advances in ICB therapy, including those targeting programmed cell death protein 1 (PD-1)/PD-ligand 1 (PD-L1) and/or cytotoxic T-lymphocyte-associated protein 4 (CTLA-4), have allowed strong enhancement of anti-tumor immune responses (Hargadon et al., 2018). Although there have been recent positive reports regarding immunotherapy, its effectiveness widely varies among individuals and tumor types. For example, in some patients with cancer, the objective response rate of anti-PD-L1/PD-1 therapy alone is only 10%–30% (Barrueto et al., 2020; Genova et al., 2022). This could be mainly attributed to the fact that current treatments specifically focus on the cancer cells rather than comprehensively addressing the tumor immune microenvironment (TIME).

Tumor cells and the TIME influence each other. Various immune cells, stromal cells, and cytokines inherently present in the TIME can interact with tumor cells. Moreover, regulation of the immune system network has complex interactions with tumor cells and crucially affects tumor development and the response to immunotherapy (Li et al., 2020a). Tumor cells make direct or indirect contact with various TIME components, with continuous and dynamic changes in the surrounding environment that allow them to evade the host immune surveillance (Mao et al., 2021; Liu et al., 2022). Additionally, the TIME is heterogeneous. The composition of tumor-infiltrating immune cells varies according to the cancer type. Therefore, it is important to elucidate the effects of multiple factors in order to accurately predict the overall effect of the TIME on the tumor immune response, which could improve survival (Morad et al., 2021). Moreover, it could inform the identification of predictive biomarkers for the efficacy of ICBs from the TIME perspective. Additionally, elucidating the mechanisms underlying the efficacy of ICBs and tumor-TIME interactions could inform individualized anti-tumor immunotherapy as well as monitoring of treatment efficacy and disease progression.

Anillin actin-binding protein (ANLN), which is a protein-coding gene, is localized on chromosome 7q14.2 and encodes 1,125 amino acid proteins. It is crucially involved in human diseases and multiple cellular functions, including cell growth, cytokinesis, mitosis, and cell migration (Xiao et al., 2020). Through interactions with cytoskeletal components, ANLN is involved in regulating tumor cell growth, invasion, and metastasis (Naydenov et al., 2021). ANLN is overexpressed in different tumor types, including breast cancer (Downs et al., 2019), lung cancer (Suzuki et al., 2005; Deng et al., 2021), pancreatic cancer (Idichi et al., 2017; Wang et al., 2019), liver cancer (Lian et al., 2018), bladder cancer (Zeng et al., 2017), and colorectal cancer (Wang et al., 2016). Moreover, it worsens the prognosis by promoting malignant tumor progression.

However, the pathogenic role of ANLN in various cancers remains unclear. Further, it remains unclear whether ANLN is involved in different tumor immune microenvironments and can influence the therapeutic response through certain common molecular mechanisms. Accordingly, through bioinformatics analysis, we aimed to elucidate the relationship of ANLN with tumor prognosis, immune infiltration, and therapeutic response in order to determine whether ANLN can be used as a biomarker for cancer screening, prognosis, individualized therapy design, and follow-up.

## Materials and methods

### Data source

**FIGURE 1:**
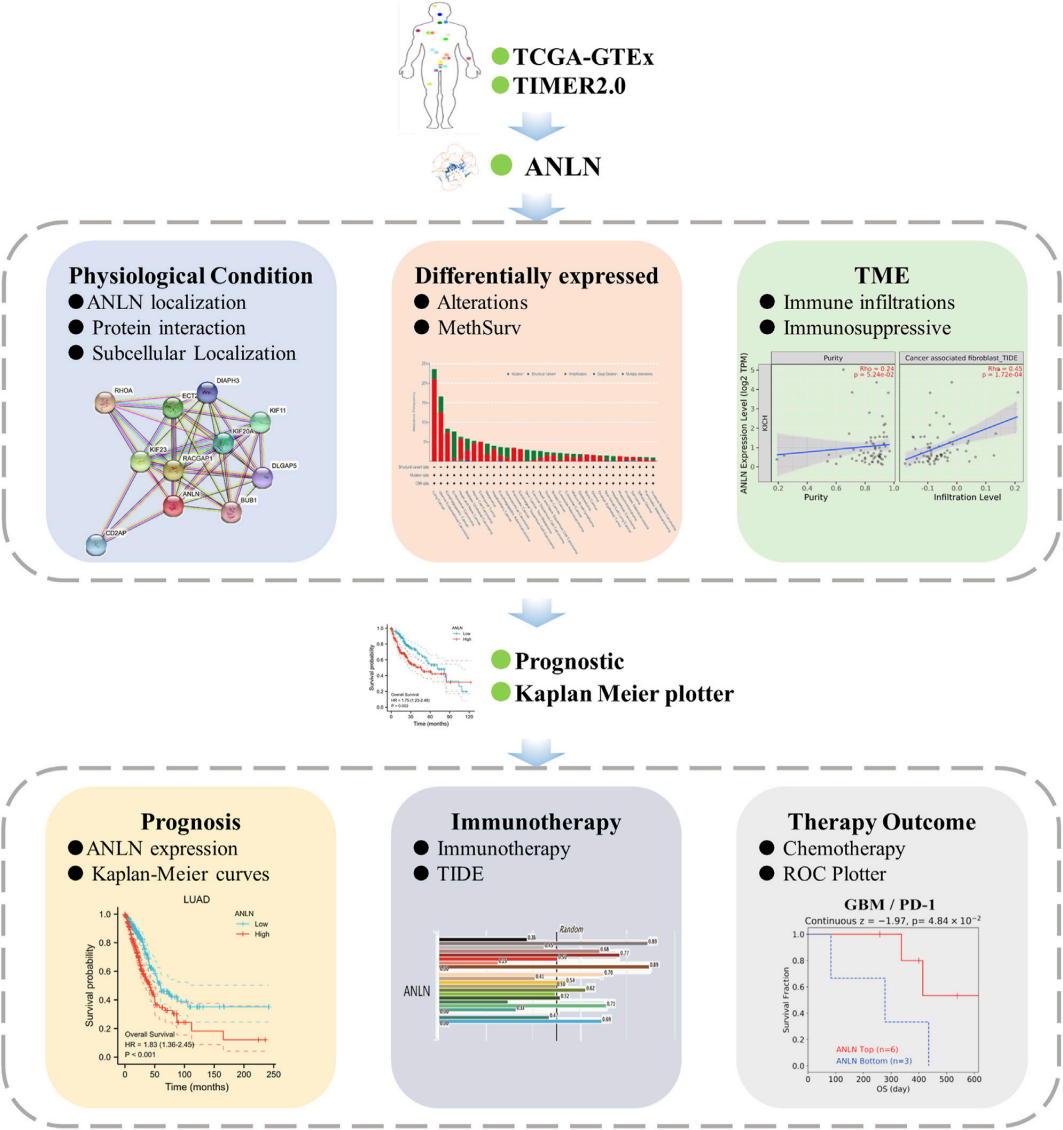
The workflow of the study.

We downloaded the clinical information and processed RNA-sequencing data of patients with 33 types of cancer from The Cancer Genome Atlas (TCGA), which is publicly available, and included 9,807 tumor tissues (Blum et al., 2018). Data regarding normal tissue expression were obtained from the Genotype-Tissue Expression (GTEx, gtexportal.org/home/) project (Carithers and Moore, 2015). We used a database of novel junctions identified in TCGA and GTEx to determine the ANLN expression in various types of human cancers and adjacent normal tissues. The UALCAN portal allows the evaluation of epigenetic regulation of gene expression through promoter methylation as well as pan-cancer gene expression analysis (Chandrashekar et al., 2017). We used the “CPTAC Analysis” module provided by the UALCAN portal to compare ANLN expression levels between normal and tumor tissues. Figure 1 shows the study flowchart.

### The physiological condition and protein interaction of anillin actin-binding protein

The Human Protein Atlas (proteinatlas.org/) web server can map all human proteins using an integration of various omics technologies, including antibody-based imaging, mass spectrometry-based proteomics, transcriptomics, and systems biology (Uhlen et al., 2015; Thul et al., 2017; Uhlen et al., 2017; Karlsson et al., 2021). PROTTER (wlab.ethz.ch/protter/start/) is an online tool for protein-form visualization as well as annotation and prediction of sequence features with experimental proteomics evidence (Omasits et al., 2014). String (cn.string-db.org/) is an online database of known and predicted protein-protein interaction (PPI) networks (confidence score cutoff = 0.7) (Szklarczyk et al., 2021).

### Differential anillin actin-binding protein expression in tumor pathological stages and prognostic analysis

Kaplan Meier plotter (kmplot.com/analysis/) is an online tool for analyzing the association of ANLN expression and genetic alterations with therapeutic outcomes in different tumors (Lanczky and Gyorffy, 2021). Based on the median ANLN expression levels, patient samples were allocated to the high and low ANLN expression groups.

The Gene Expression Profiling Interactive Analysis (GEPIA) (gepia.cancer-pku.cn/index.html) is a novel interactive web server that provides customizable functions, including tumor/normal differential expression analysis, profiling according to cancer types or pathological stages, and patient survival analysis. GEPIA uses a standard processing pipeline to analyze the RNA sequencing expression data of 9,736 tumors and 8,587 normal samples from the TCGA and the GTEx projects (Tang et al., 2017). The GEPIA database was used to evaluate differential ANLN expression between tumor tissues and para-tumor tissues, as well as ANLN expression in different tumor pathological stages.

### Tumor DNA methylation and gene mutation characteristics

MethSurv (version MethSurv^©^2017, biit.cs.ut.ee/methsurv/), which is a web tool for survival analysis based on the CpG methylation patterns (Modhukur et al., 2018), was used to analyze DNA methylation sites of ANLN based on the TCGA data. Moreover, we used the Kaplan-Meier test in the MethSurv database to perform overall survival (OS) analysis for each CpG site. Significance was tested using a likelihood ratio test. Density plots were used to visualize cut-off points for the dichotomy of methylation levels (Supplementary Figure S1). The plots were drawn with the β-value as the abscissa and the corresponding sample proportion as the ordinate. The numbers in red indicate the cutoff points used to group patients.

The cBioPortal for Cancer Genomics (cbioportal.org/) is an open-access, open-source resource for interactive exploration of multiple cancer genomics datasets (Cerami et al., 2012). Currently, it stores data regarding the DNA copy number, mRNA and microRNA expression, non-synonymous mutations, protein and phosphoprotein level, DNA methylation, and limited clinical information (Cerami et al., 2012). The ANLN mutation signatures in pan-tumors were investigated on cBioPortal OncoPrint using data obtained from the TCGA Pan-Cancer Atlas (PanCanAtlas) cohort.

### Analysis of tumor immune cell infiltration

Tumor IMmune Estimation Resource (TIMER2.0) (timer.- cistrome.org/), which is a web server for analyzing tumor-infiltrating immune cells using TCGA (Li et al., 2020b), was used to analyze the relationship of immune cell infiltration abundance with ANLN expression levels in different cancer types. These infiltrating immune cells include B cells, CD8^+^ T-cells, CD4^+^ T-cells, macrophages, neutrophils, dendritic cells (DCs), myeloid-derived suppressor cells (MDSCs), cancer-associated fibroblasts (CAFs), M2-tumor-associated macrophages (TAMs), and T-regulatory lymphocytes (Treg cells) *via* gene modules (Summerfield et al., 2001). Spearman’s correlation coefficients were used to analyze the correlation between ANLN and immune cell infiltration abundance. Statistical significance was set at *p* < 0.05. Data were visualized in GraphPad Prism Software (Version 8.0.0 for Windows). A heatmap was used to visualize the correlation of immune cell infiltration abundance with ANLN expression in 33 cancer types in the TCGA.

### Analysis of the correlation between the tumor treatment response and anillin actin-binding protein expression

The Tumor Immune Dysfunction and Exclusion (TIDE) database (tide.dfci.harvard.edu) comprises omics data regarding over 33K samples obtained from 188 tumor cohorts in public databases, 998 tumors from 12 clinical studies on ICBs, and eight CRISPR screens. It provides a computational method for modeling the two primary mechanisms of tumor immune evasion (Jiang et al., 2018; Fu et al., 2020). Gene prioritization of ANLN was assessed based on the treatment response to ICBs while knockout phenotypes were identified through CRISPR screening. The z-score in Cox-PH regression was used to assess the effect of gene expression on survival in the ICB-treatment cohort. CRISPR screening was used to assess the role of knockout-mediated lymphocyte-induced tumor death in cancer models. Based on the TIDE biomarker assessment module, we examined the overall predictive utility of ANLN for treatment response outcomes and OS across cancer types; further, we compared ANLN with standardized biomarkers for tumor immune response.

The ROC Plotter (rocplot.org/) is the first online transcriptome-level validation tool for predictive biomarkers. It can link gene expression to treatment response in patients with breast, ovarian, colorectal, and glioblastoma cancer (Fekete and Gyorffy, 2019). We used the ROC plotter to assess the predictive utility of ANLN expression for treatment response in patients with cancer.

### Statistical analysis

All expression data were normalized and log-transformed using log2 (data + 1). R software (V3.8.3) was used to compare differential ANLN expression between tumor and normal tissues. Between-group comparisons were performed using t-tests (ns, *p* ≥ 0.05; *, *p* < 0.05; **, *p* < 0.01; ***, *p* < 0.001). The R package [ggplot2 (version 3.3.3)] was used for visualization. The Wilcoxon rank-sum test was used to examine the relationship of ANLN expression with pathologic/cytogenetic features. For survival analysis, the hazard ratio (HR) and 95% confidence intervals (CI) were derived from Cox proportional hazards models. The *p*-value was determined using Kaplan-Meier analysis with the log-rank test. Statistical significance was set at *p* < 0.05.

## Results

### Topology, localization, expression profile, and protein interaction network of anillin actin-binding protein

**FIGURE 2:**
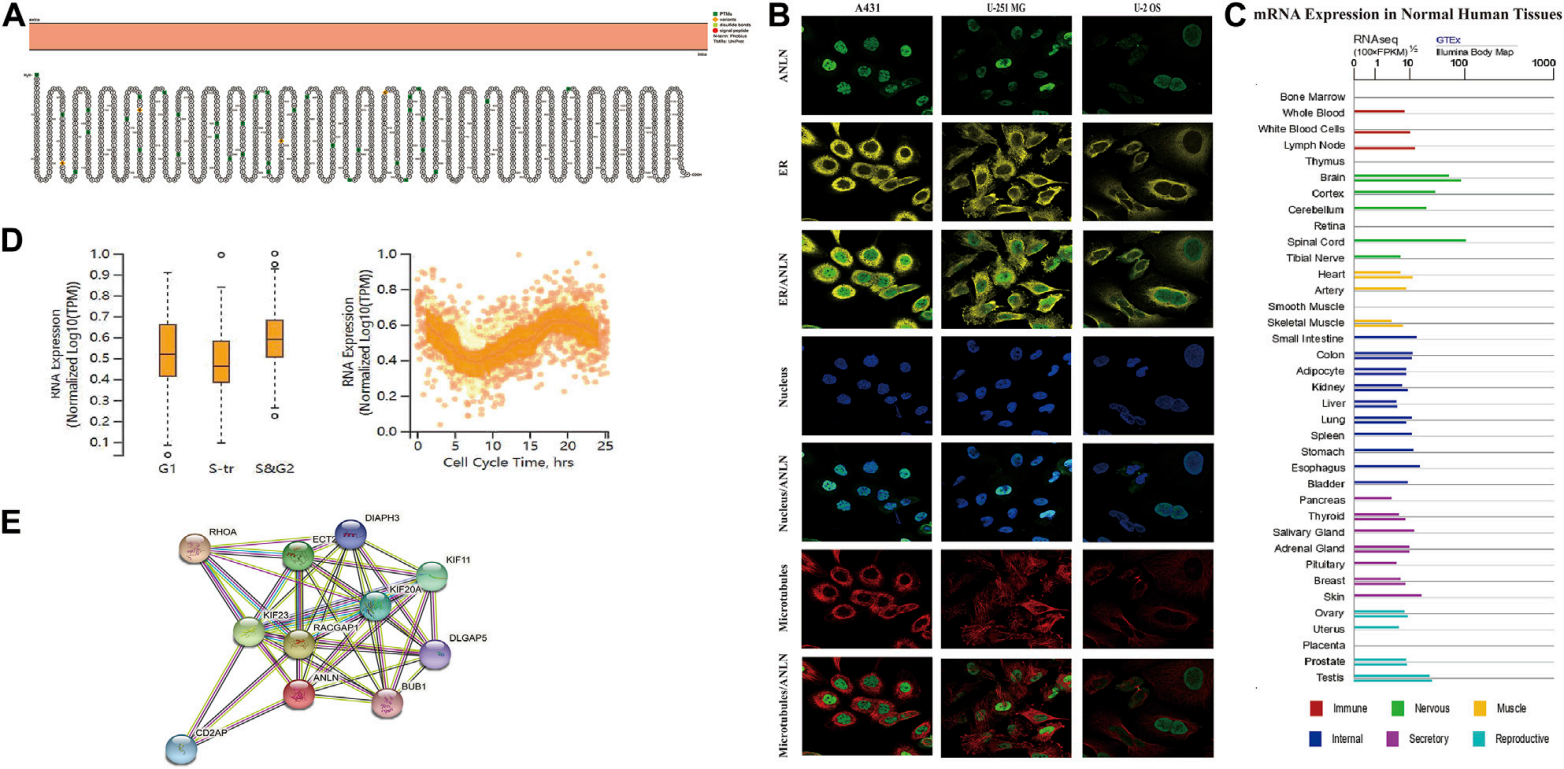
Topology, localization, expression profile, and protein interaction network of ANLN proteins. **(A)** Topology of ANLN proteins showing intracellular membrane localization. **(B)** Immunofluorescence staining of the subcellular distribution of ANLN in the nucleus, endoplasmic reticulum, and microtubules of A-431, U-2 OS, and U-251 MG was adopted from the HPA database. **(C)** Bar graphs of ANLN mRNA expression in various normal human tissues from the TCGA_GTEx database. **(D)** The plot of single-cell RNA sequencing data from the U-2 OS cell line shows a correlation between ANLN mRNA expression and cell cycle progression. **(E)** Protein interactions network of ANLN.

Under physiological conditions, the topology of ANLN proteins indicated intracellular membrane localization (Figure 2A). To characterize the intracellular localization of ANLN, we used an indirect immunofluorescence assay to investigate the distribution of ANLN in the endoplasmic reticulum (ER), nucleus, and microtubules of A-431, U-2 OS, and U-251 MG cells. The Human Protein Atlas database showed that ANLN localization was mainly distributed in the nucleus, but not in the ER or microtubules, of these cells (Figure 2B). In addition, ANLN mRNA expressions occur in various normal human tissues, including the immune, visceral, nervous, endocrine, muscle, and reproductive tissues (Figure 2C). Based on the fluorescent ubiquitination-based cell cycle indicator system, single-cell RNA sequencing analysis of U-2 OS cells revealed an association of increased ANLN RNA expression with cell cycle progression (Figure 2D). Moreover, ANLN expression was correlated with the cell cycle progression in the G1, S, and S/G2 phases (Figure 2D). PPI analysis revealed that proteins related to ANLN included RHOA, RACGAP1, KIF23, ECT2, KIF11, KIF20A, CD2AP, DIAPH3, DLGAP5, and BUB1 (Figure 2E).

### Anillin actin-binding protein expression in pancytopenia

**FIGURE 3:**
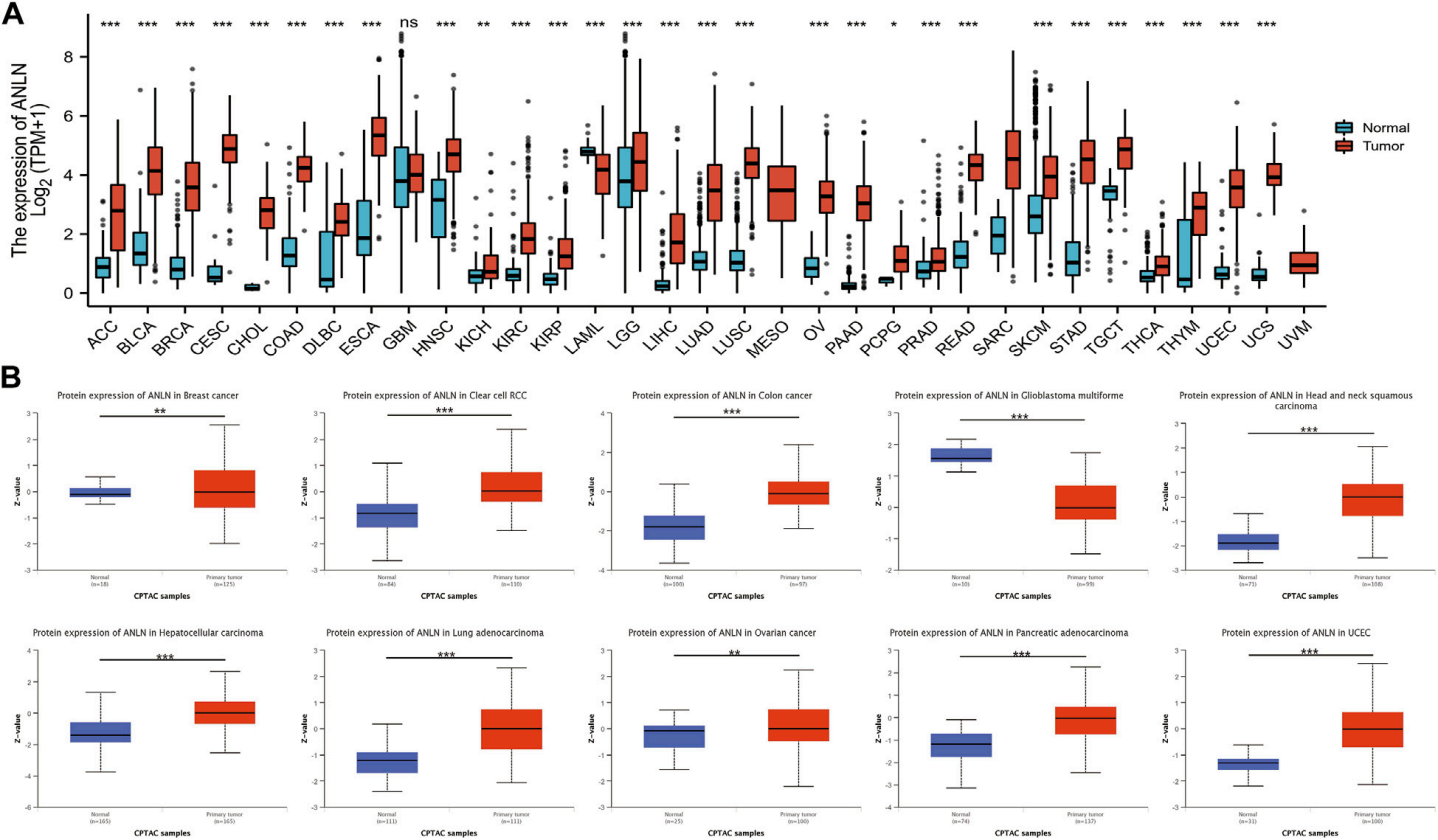
Upregulation of ANLN mRNA expression in pan-cancer. **(A)** Expression levels of ANLN in TCGA_GTEx database in different cancer types. Log2 (TPM + 1) was used for log scale. **(B)** Expression levels of ANLN protein in normal and different tumor tissues from the CPTAC database. *p < 0.05, **p < 0.01, and ***p < 0.001.

Based on the TCGA_GTEx database, there was significantly higher ANLN expression in tumor tissues than in adjacent normal tissues in adrenocortical carcinoma (ACC), bladder urothelial carcinoma (BLCA), breast invasive carcinoma (BRCA), cervical squamous cell carcinoma (CESC), cholangiocarcinoma (CHOL), colon adenocarcinoma (COAD), lymphoid neoplasm diffuse large B-cell lymphoma (DLBC), esophageal carcinoma (ESCA), head and neck squamous cell carcinoma (HNSC), kidney chromophobe (KICH), kidney renal clear cell carcinoma (KIRC), kidney renal papillary cell carcinoma (KIRP), brain lower grade glioma (LGG), liver hepatocellular carcinoma (LIHC), lung adenocarcinoma (LUAD), lung squamous cell carcinoma (LUSC), ovarian serous cystadenocarcinoma (OV), pancreatic adenocarcinoma (PAAD), pheochromocytoma and paraganglioma (PCPG), prostate adenocarcinoma (PRAD), rectum adenocarcinoma (READ), skin cutaneous melanoma (SKCM), stomach adenocarcinoma (STAD), testicular germ cell tumors (TGCT), thyroid carcinoma (THCA), thymoma (THYM), uterine corpus endometrial carcinoma (UCEC), and uterine carcinosarcoma (UCS) (Figure 3A). Further analysis using the “CPTAC Analysis” confirmed ANLN overexpression in BRCA, renal cell carcinoma, COAD, HNSC, LIHC, LUAD, OV, PAAD, and UCEC tumors compared with in normal tissues; however, there was low ANLN expression in the glioblastoma multiforme (Figure 3B).

### Correlation of anillin actin-binding protein with pathological stage and prognosis of pan-cancer

**FIGURE 4:**
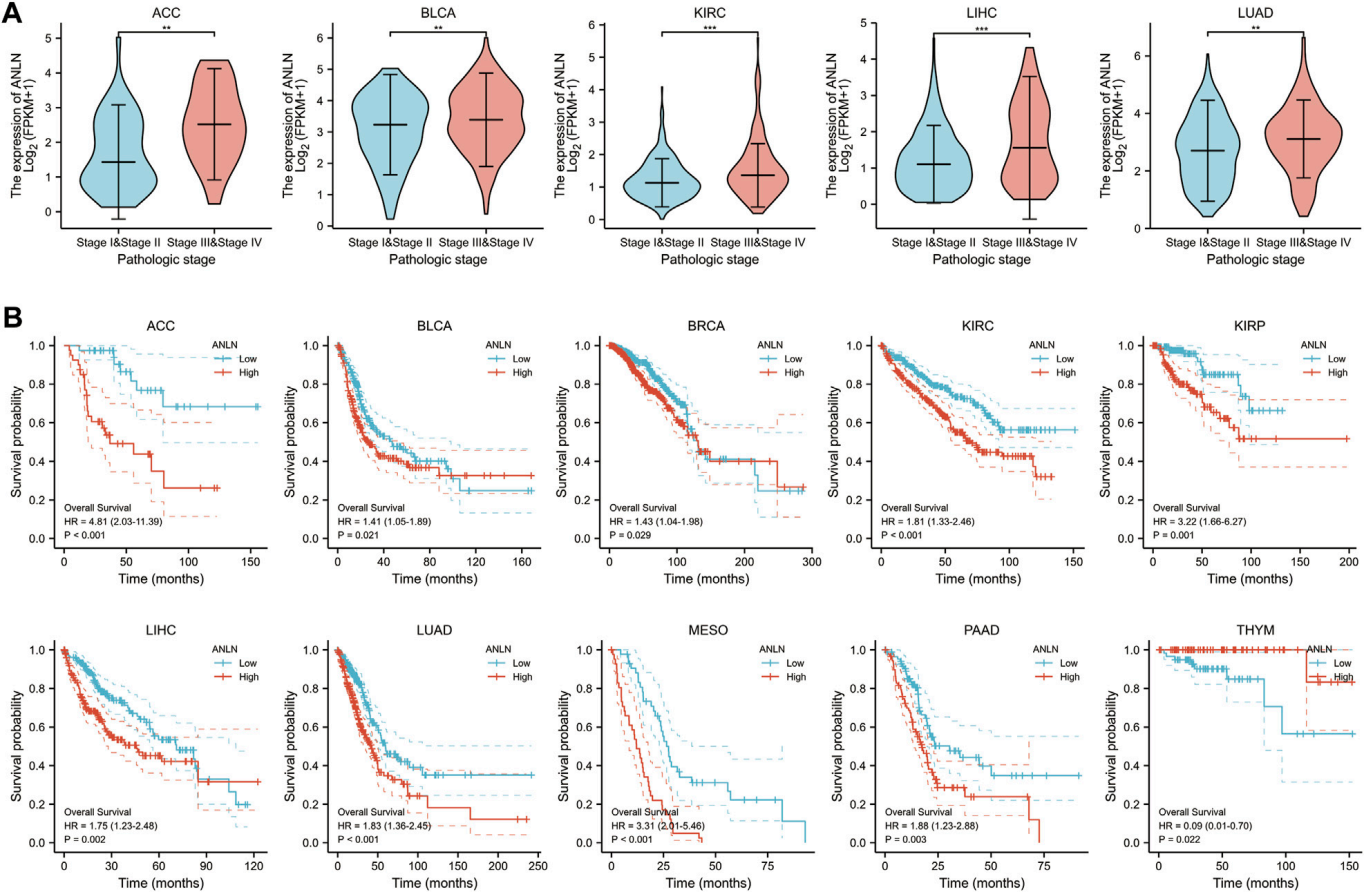
Correlation of ANLN expression with pathological stage and prognosis of tumors. **(A)** Correlation of ANLN expression with pathological stages of ACC, BLCA, KIRC, LIHC, and LUAD; **(B)** Correlation of ANLN expression with a prognosis of patients with ACC, BLCA, BRCA, KIRC, KIRP, LIHC, LUAD, MESO, PAAD, and THYM. *p < 0.05, **p < 0.01, and ***p < 0.001.

To investigate the relationship between ANLN expression and the clinicopathological features of pan-cancer, we evaluated ANLN expression in patients with pathological stages I, II, III, and IV cancers. The TCGA database showed that ANLN expression was significantly upregulated in ACC, BLCA, KIRC, LIHC, and LUAD with advanced pathological stages (Figure 4A). In pan-cancer prognostic analysis, Kaplan-Meier survival curves revealed a significant association of upregulated ANLN expression with poor OS in ACC, BLCA, BRCA, KIRC, KIRP, LIHC, LUAD, mesothelioma (MESO), and PAAD; contrastingly, low ANLN expression was associated with poor OS in THYM (Figure 4B).

### DNA methylation and mutational features of anillin actin-binding protein in pan-cancer

**FIGURE 5:**
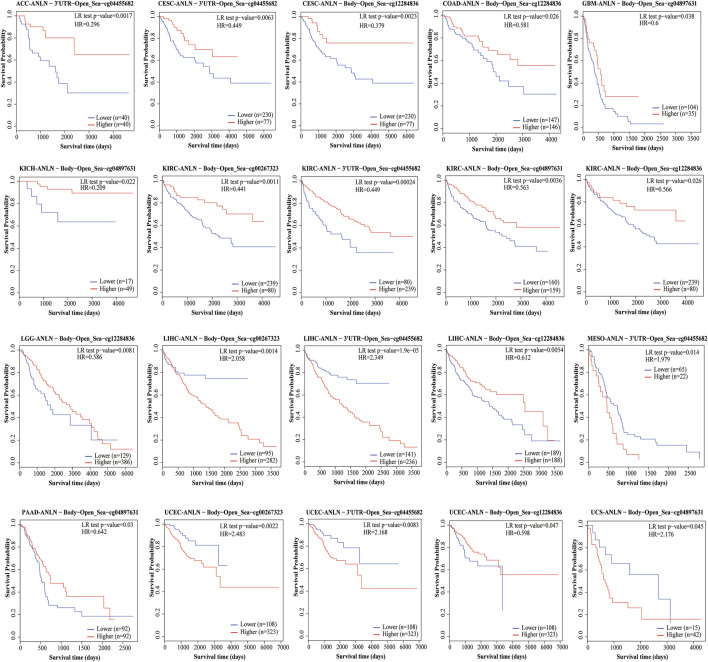
The Kaplan-Meier survival of the promoter methylation of ANLN.

DNA methylation directly affects cancer development and progression. We used the MethSurv to investigate DNA methylation of ANLN in terms of the prognostic value of each CpG site in cancer. Prognosis was correlated with the methylation level of four CpG sites, i.e., cg00267323, cg04455682, cg04897631, and cg12284836 (*p* < 0.05) (Table 1). Patients with low ANLN methylation of CpG site cg12284836 had a worse OS than patients with high ANLN methylation in this site for CESC, COAD, KIRC, LGG, LIHC, and UCEC (Figure 5).

**TABLE 1:** Effect of hypermethylation level on prognosis in cancer.

CpG	Cancer	HR	CI	*p*-value	Best-split
cg04455682	ACC	0.296	(0.133–0.658)	0.002	median
cg04455682	CESC	0.449	(0.241–0.838)	0.006	q75
cg12284836	CESC	0.379	(0.188–0.763)	0.002	q75
cg12284836	COAD	0.581	(0.357–0.944)	0.026	median
cg04897631	GBM	0.600	(0.362–0.995)	0.038	q75
cg04897631	KICH	0.209	(0.056–0.778)	0.022	q25
cg00267323	KIRC	0.441	(0.258–0.754)	0.001	q75
cg04455682	KIRC	0.449	(0.299–0.674)	0.000	q25
cg04897631	KIRC	0.563	(0.379–0.834)	0.004	median
cg12284836	KIRC	0.566	(0.332–0.964)	0.026	q75
cg12284836	LGG	0.586	(0.400–0.858)	0.008	q25
cg00267323	LIHC	2.058	(1.276–3.319)	0.001	q25
cg04455682	LIHC	2.349	(1.543–3.574)	0.000	mean
cg12284836	LIHC	0.612	(0.431–0.869)	0.005	median
cg04455682	MESO	1.979	(1.174–3.336)	0.014	q75
cg04897631	PAAD	0.642	(0.430–0.960)	0.030	median
cg00267323	UCEC	2.483	(1.306–4.721)	0.002	q25
cg04455682	UCEC	2.168	(1.164–4.039)	0.008	q25
cg12284836	UCEC	0.598	(0.366–0.976)	0.047	q25
cg04897631	UCS	2.176	(0.971–4.878)	0.045	q25

**FIGURE 6:**
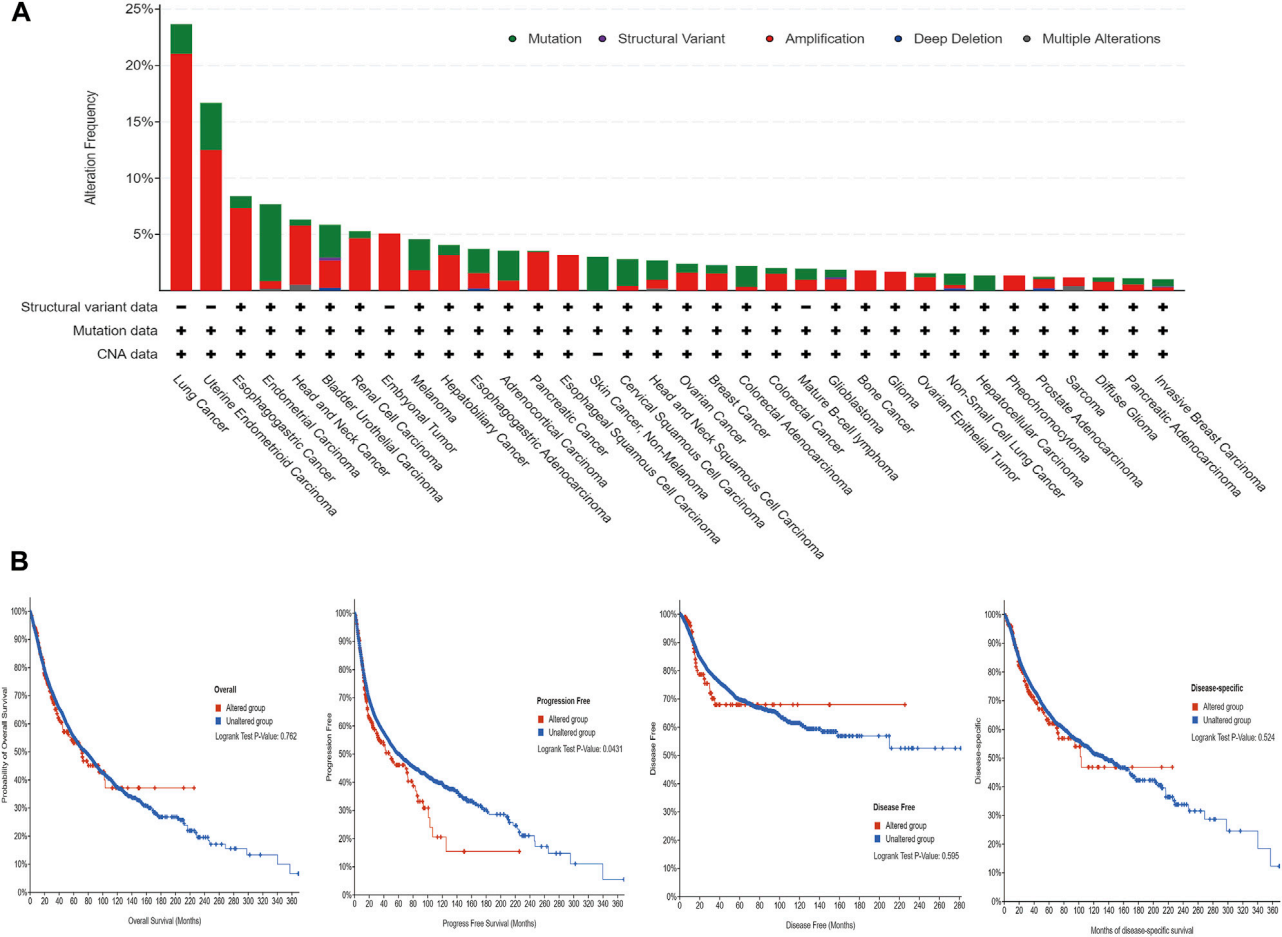
The mutational characteristics of ANLN in cancer. **(A)** The frequency of alterations in different types of mutations was examined using the cBioPortal database. **(B)** The effect of ANLN mutation status on overall survival (OS), disease-specific survival (DSS), disease-free survival (DFS), and progression-free survival (PFS) of cancer patients was investigated using the cBioPortal database.

The cBioPortal (TCGA, Pan-Cancer Atlas) database showed that lung cancer had the highest frequency of ANLN mutation (≈ 23.68%) (Figure 6A). As shown in Figure 6A, ANLN amplification and mutation were the most common alteration type. Moreover, among the patients with tumors, patients with ANLN mutations had worse progression-free survival (PFS); however, there were no significant differences in OS, disease-free survival, and disease-specific survival (DSS) (Figure 6B).

### Correlation of anillin actin-binding protein with tumor immunity

**FIGURE 7:**
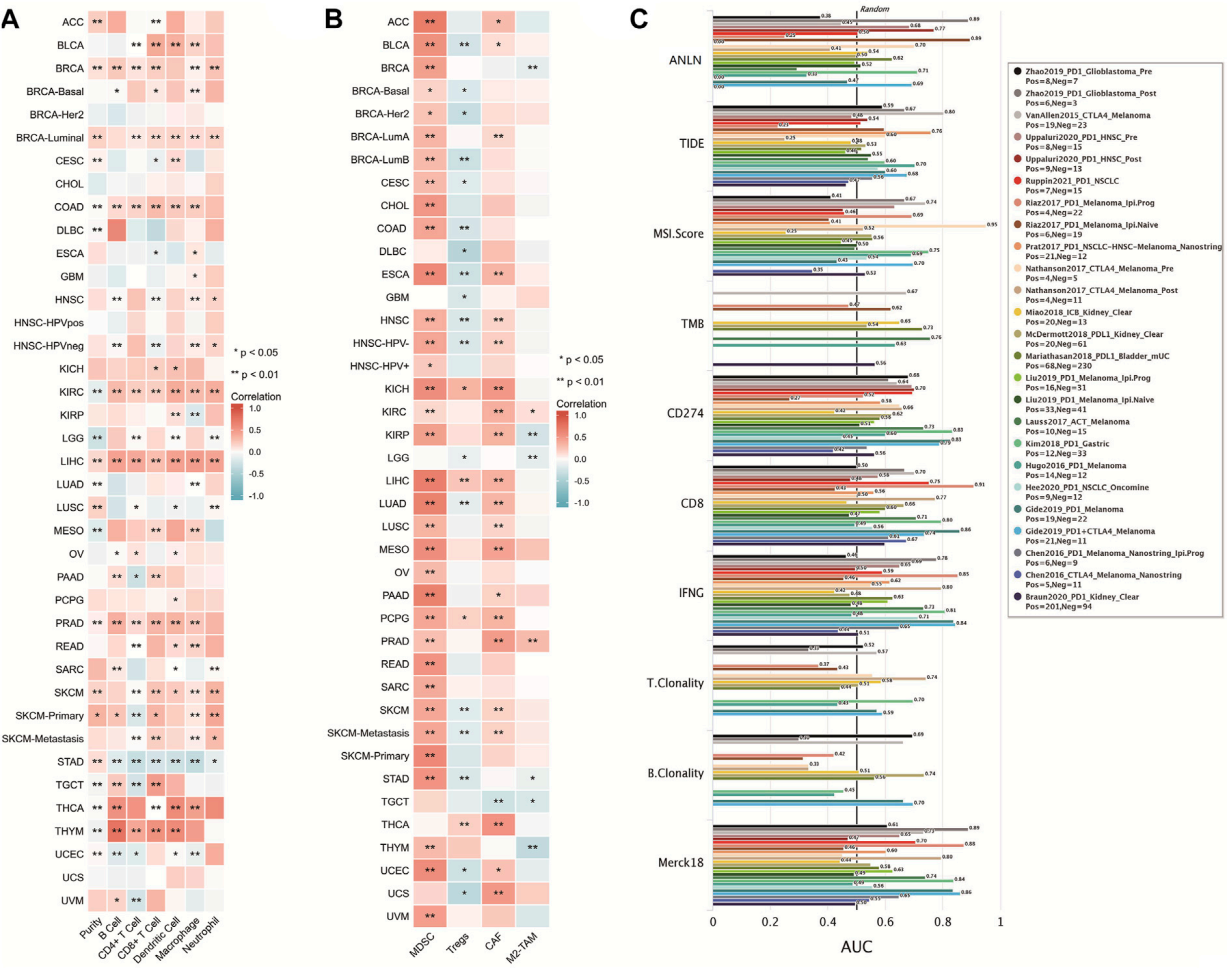
Correlation of ANLN with tumor immune cell infiltration. **(A,B)** Heat map showing a correlation between ANLN protein and infiltration of six types of immune cells and four types of immunosuppressive cells in multiple tumors; **(C)** Comparison of ANLN with other biomarkers for efficacy prediction of immunodetectable point-of-care inhibitors.

Based on the TCGA database, ANLN expression in COAD, KIRC, LIHC, and PRAD showed a significant positive correlation with infiltration of six immune cells (B cells, CD8^+^ T-cells, CD4^+^ T-cells, macrophages, neutrophils, and DCs) (*p* < 0.05; Figure 7A). Additionally, we assessed the correlation of ANLN expression with infiltration of four immunosuppressive cells (MDSCs, CAFs, M2-TAMs, and Treg cells), which promote T-cell rejection. ANLN expression was positively correlated with myeloid-derived suppressor cell infiltration in ACC, BLCA, BRCA, BRCA-Basal, BRCA-Her2, BRCA-LumA, BRCA-Lumb, CESC, CHOL, COAD, ESCA, HNSC, HNSC-HPV−, HNSC-HPV+, KICH, KIRC, KIRP, LIHC, LUAD, LUSC, MESO, OV, PAAD, PCPG, PRAD, READ, SARC, SKCM, SKCM-Metastasis, SKCM-Primary, STAD, THYM, UCEC, and uveal melanoma (*p* < 0.05). ANLN expression was positively correlated with Tregs infiltration in KICH, LIHC, PCPG, and THCA (*p* < 0.05); ANLN expression was positively correlated with CAF infiltration in ACC, BLCA, BRCA-LumA, ESCA, HNSC, HNSC-HPV-, KICH, KIRC, KIRP, LIHC, LUAD, LUSC, MESO, PAAD, PCPG, PRAD, SKCM, SKCM-Metastasis, THCA, UCEC, and UCS (*p* < 0.05). ANLN expression was positively correlated with M2-TAM infiltration in KIRC and PRAD (*p* < 0.05) (Figure 7B).

We compared the predictive utility of ANLN and other standardized biomarkers for the efficacy of ICB. Among the ICB subgroups, 10 had ANLN-AUC > 0.5 (Figure 7C). ANLN showed a higher predictive value than TMB, T.Clonality, and B.Clonality.

### Anillin actin-binding protein is strongly correlated with tumor treatment outcomes

**FIGURE 8:**
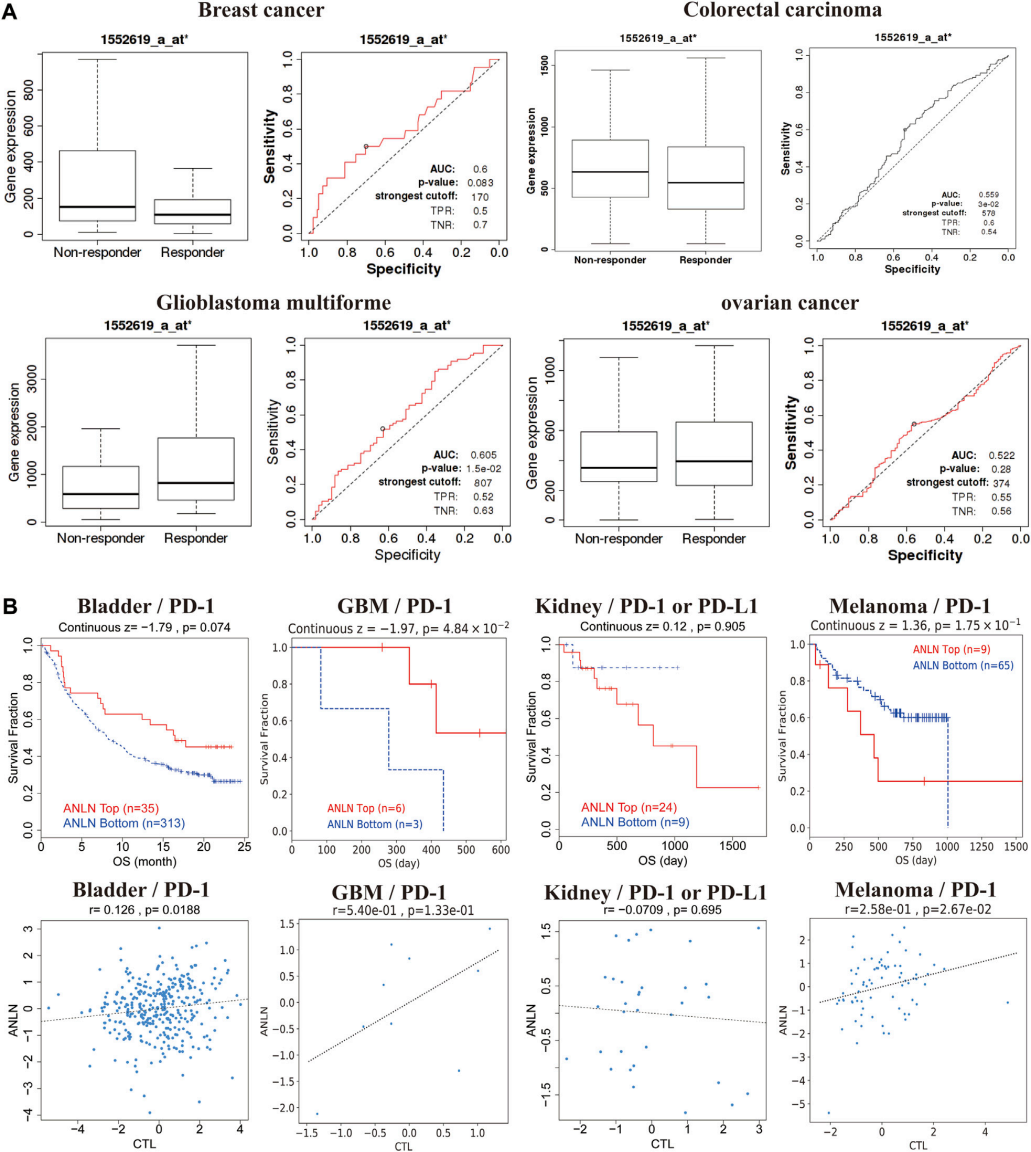
ANLN expression correlates with treatment response in multiple cancer types. **(A)** ROC plot of the association between ANLN expression and chemotherapy response in breast, brain, colorectal, and ovarian cancer cohorts. **(B)** Kaplan-Meier curves (upper panel) as a measure of immunotherapy response between cancer cohorts with high and low ANLN expression. The graph below shows the correlation between ANLN expression and cytotoxic T-cell levels.

We evaluated the effect of ANLN expression on chemotherapy response in a clinical cancer cohort. Patients with breast and colorectal cancer who presented high ANLN expression showed resistance to chemotherapy. Contrastingly, among patients with glioblastoma and ovarian cancer, patients with high ANLN expression showed greater benefits from chemotherapy than those with low ANLN expression (Figure 8A). Additionally, high ANLN expression was associated with clinical benefits derived from ICB therapy (PD-1), and thus longer OS, in patients with bladder cancer and glioblastoma. Contrastingly, in patients with kidney cancer and melanoma, low ANLN expression was associated with clinical benefits derived from ICB therapy (PD-L1 or PD-1), and thus longer OS (Figure 8B). In patients with bladder cancer and melanoma, high ANLN expression was positively correlated with CTL levels (Figure 8B).

## Discussion

Recently, there have been increasing studies using Pan-Cancer Analysis of Whole Genomes to reveal genetic mutations, RNA changes, and oncogenes associated with cancer development and progression. Additionally, pan-cancer analysis has revealed common aspects among cancer types regarding key driver gene mutations, tumor-associated signaling pathway activation, immune escape, and microbial features. This suggests that the same therapeutic strategy can be applied for common features in different cancer types (Chen et al., 2021). Therefore, Pan-Cancer analysis may provide novel biomarkers for early diagnosis, prognosis, and treatment efficacy in patients with cancer.

ANLN, which is a key protein in cell division, can combine with GTP-Rho, actin, and cytokinin. ANLN is crucially involved in cell proliferation, differentiation, adhesion, migration, apoptosis, and cycle progression (Naydenov et al., 2021). Abnormal ANLN expression causes dysregulation of cell division and is crucially involved in cancer development, cancer cell proliferation, and metastasis. We found that ANLN mRNA expression was positively correlated with cell cycle progression; moreover, most tumors showed high ANLN expression, which was significantly correlated with poor prognosis and tumor progression. In bladder cancer, there was ANLN overexpression, which could promote tumor cell proliferation, migration, and infiltration; moreover, this was confirmed *in vitro* experiments. Nuclear ANLN overexpression is significantly correlated with tumor stage, histological grade, and tumor invasion; additionally, it is an independent risk factor for DSS (Liang et al., 2015; Zeng et al., 2017). In gastric cancer, ANLN can promote cell proliferation by activating the Wnt/β-catenin signaling pathway (Pandi et al., 2014). Li et al. (2020c) reported that the CDK1-PLK1-SGOL2-ANLN signaling axis mediating abnormal cell cycle division may be crucially involved in the development of hepatocellular carcinoma (HCC). Moreover, tumor tissues present with high ANLN expression and decreased ANLN methylation in HCC. Further, ANLN silencing may inhibit HCC cell proliferation in the G2/M phase as well as their migration and invasion abilities (Zhang et al., 2021). Contrastingly, the metastatic foci of HCC showed significantly higher cytosolic ANLN expression than the primary tumor tissue; additionally, high cytosolic ANLN expression was an independent risk factor for 5-year OS after hepatic resection. This study suggested that ANLN could be a potential therapeutic target and prognostic biological marker for HCC (Zhang et al., 2021). In gastric cancer, decreased ANLN expression affects downstream DNA damage repair-related pathways, resulting in more irreparable DNA damage in S-phase cells and eventually apoptosis (Wei et al., 2020). Patients with non-small cell lung cancer present with the highest ANLN expression. Inhibiting ANLN protein expression through siRNA could inhibit tumor cell growth and migration as well as induce tumor cell apoptosis (Suzuki et al., 2005). In pancreatic cancer cells, ANLN deficiency promotes miR218-5p expression, which results in apoptosis (Liu et al., 2015). Additionally, ANLN is a key modulator of inducing PI3K/Akt signaling, which suggests that ANLN is indirectly associated with apoptosis induction (Dai et al., 2019). In HNSC, ANLN is overexpressed in tumor tissues, with ANLN knockdown affecting tumor cell proliferation, migration, and invasion. Moreover, exosome-derived ANLN promotes cell differentiation and macrophage polarization via the PTEN/PI3K/Akt signaling pathway, which subsequently stimulates tumor growth. Taken together, ANLN is an independent prognostic factor for patients with HNSC (Guo et al., 2021). Our study used bioinformatics methods to further demonstrate the correlation of ANLN with tumor progression and prognosis, which provides a theoretical basis for the use of ANLN as a potential novel target for biotherapy and a biomarker for tumor surveillance.

The TIME facilitates tumor growth and function by providing a relatively stable living environment for cancer cells to continuously proliferate without interference, which enhances the tumor malignancy potential. Additionally, it contains numerous immune and immunosuppressive cells, including M2-TAMs, CAFs, Tregs, and MDSCs, which can suppress the production, activation, and infiltration of cytotoxic T-cells as well as promote tumor immune evasion, tumor growth, metastasis, and treatment resistance (Liu and Cao, 2016; Monteran and Erez, 2019). The TIME and tumor immune escape are associated with cancer prognosis and treatment (Wu and Dai, 2017). Regarding tumor immune escape mechanisms, tumor immune cell infiltration causes T-cell dysfunction, which promotes tumor evasion of host immune system surveillance as well as facilitates tumor progression, invasion, metastasis, and treatment resistance (Yu et al., 2009; Liu et al., 2022). Another tumor immune escape mechanism is T-cell rejection. Here, CAFs, Tregs, M2-TAMs, and MDSCs in the tumor microenvironment, which act as immunosuppressive cells, block immune cell infiltration (Man et al., 2013; Komohara et al., 2016). Based on the TCGA database, we analyzed the relationship of ANLN with tumor immune cell infiltration. ANLN showed a significant positive correlation with tumor immune cell infiltration only in COAD, KIRC, LIHC, and PRAD, indicating that ANLN could enhance tumor immune infiltration through T-cell dysfunction in these cancer types. Furthermore, ANLN expression was strongly correlated with the levels of immunosuppressive cells in almost all cancer types. This suggests that T-cell rejection is the main mechanism through which ANLN regulates immune cell tumor escape, tumor promotion, and metastasis.

DNA methylation modifications affect a range of biological processes such as eukaryotic cell growth, differentiation, and transformation mechanisms by regulating gene expression. DNA methylation dysregulation causes multiple diseases (Wagner, 2022) and is considered a key event in cancer development and progression, including altered DNA methylation and dysregulation of gene expression in tumor-related genes (Wang J. et al., 2021). The expression of aberrantly methylated genes is associated with the degree of tumor differentiation, TNM stage, and poor prognosis (Skvortsova et al., 2019). Therefore, we investigated the relationship of ANLN methylation levels with prognosis in patients with cancer. We found that prognosis was correlated with the methylation level of four CpG sites; namely, cg00267323, cg04455682, cg04897631, and cg12284836.

Although genetic alterations in tumors are common, premalignant genetic changes are more likely to initiate and promote cancer development. The accumulation of genetic alterations drives the progression of normal cells through hyperplastic and dysplastic stages to invasive cancer and, ultimately, metastatic disease (Garnis et al., 2004). Therefore, analyzing genetic alterations in known oncogenes could further elucidate their role in cancer progression. In our study, lung cancer showed the highest frequency of ANLN alterations (≈ 23.68%); moreover, amplification and mutation were the most common genetic alterations. In addition, patients with altered ANLN genes showed worse PFS. Nonetheless, further experimental studies are warranted to reveal the mechanisms through which ANLN gene alterations are involved in tumor development.

There has been increasing research interest in immune replacement therapy regimens based on PD-1 and PD-L1 inhibitors. PD-1 and PD-L1-based immunotherapy can effectively treat various cancers and improve prognosis (Makuku et al., 2021). We found that high ANLN expression was associated with the clinical benefits of ICB therapy (PD-1) in patients with bladder cancer and glioblastoma, while low ANLN expression was associated with the clinical benefits of ICB therapy (PD-L1 or PD-1) in patients with kidney cancer and melanoma. This suggested that ANLN is crucially involved in tumor-mediated immune escape. Further, regarding the relevance of ANLN in oncologic chemotherapy, in patients with breast and colorectal cancers, ANLN expression was negatively correlated with sensitivity to chemotherapeutic drugs. Patients with breast cancer showed high ANLN expression in tumor tissues; moreover, ANLN downregulation affected the tumor cell cycle and promoted apoptosis while inhibiting tumor cell proliferation, migration, and invasion. Therapy targeting ANLN could provide a similar therapeutic effect as chemotherapy on breast cancer (Wang Z. et al., 2021). However, the mechanism through which ANLN mediates resistance to chemotherapeutic agents should be further investigated.

## Conclusion

In summary, our findings showed that ANLN is strongly associated with the prognosis, immune cell infiltration, gene mutations, and tumor treatment in patients with cancer. These findings provide further insight into the role of ANLN in tumorigenesis from an overall perspective. Furthermore, ANLN could be an onco-immunological biomarker and could serve as a hallmark for tumor screening, prognosis, individualized treatment design, and follow-up.

## Data Availability

The original contributions presented in the study are included in the article/Supplementary Material, further inquiries can be directed to the corresponding authors.

## Supplementary material

Supplementary Figure S1 The density plots were used to visualize cut-off points for the dichotomy of methylation levels in patients.
